# What Are the Molecular Requirements for Protein Sliding along DNA?

**DOI:** 10.1021/acs.jpcb.1c00757

**Published:** 2021-03-23

**Authors:** Lavi S. Bigman, Harry M. Greenblatt, Yaakov Levy

**Affiliations:** Department of Chemical and Structural Biology, Weizmann Institute of Science, Rehovot 76100, Israel

## Abstract

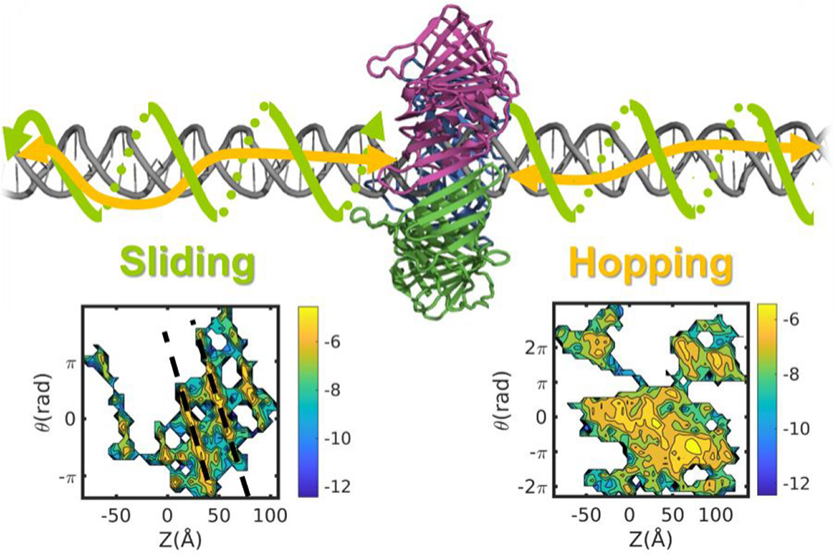

DNA-binding proteins rely on linear diffusion along the longitudinal DNA axis, supported by their nonspecific electrostatic affinity for DNA, to search for their target recognition sites. One may therefore expect that the ability to engage in linear diffusion along DNA is universal to all DNA-binding proteins, with the detailed biophysical characteristics of that diffusion differing between proteins depending on their structures and functions. One key question is whether the linear diffusion mechanism is defined by translation coupled with rotation, a mechanism that is often termed sliding. We conduct coarse-grained and atomistic molecular dynamics simulations to investigate the minimal requirements for protein sliding along DNA. We show that coupling, while widespread, is not universal. DNA-binding proteins that slide along DNA transition to uncoupled translation–rotation (i.e., hopping) at higher salt concentrations. Furthermore, and consistently with experimental reports, we find that the sliding mechanism is the less dominant mechanism for some DNA-binding proteins, even at low salt concentrations. In particular, the toroidal PCNA protein is shown to follow the hopping rather than the sliding mechanism.

## Introduction

The transportation of biomolecules via diffusion is essential to many cellular processes. The most widespread form of cellular transportation involves the three-dimensional (3D) translational diffusion of molecules in the cytoplasm or through membranes. Proper cellular functioning also demands diffusion in lower dimensional spaces. For example, proteins translationally diffuse on two-dimensional surfaces (2D) such as membranes. Furthermore, several cellular functions are governed by linear diffusion in one-dimensional (1D) space. Many proteins were reported to diffuse linearly along the elongated axis of double-helical DNA.^1−3^ Also, single-stranded DNA molecules were shown to diffuse linearly along the surface of their binding protein partners.^4,5^ Similarly, linear translational diffusion was also shown experimentally and computationally at protein–protein interfaces. A common example of 1D diffusion at the protein–protein interfaces involves microtubule-binding proteins translocating along the longitudinal axis of the microtubule protofilament.^6−9^ Furthermore, 1D translational diffusion was observed along the interfaces of dimeric coiled-coil protein complexes.^10^

The diffusion mechanisms of biomolecules depend on the dimensionality of the space, and consequently diffusion differs in 1D, 2D, and 3D spaces. Furthermore, diffusion in 1D, 2D, or 3D may depend on various molecular characteristics of the diffusing proteins as well as the medium. For example, linear diffusion of proteins along microtubules is different for intrinsically disordered proteins and globular proteins.^11^ Modifying microtubules post-translationally (e.g., polyglutamylation or polyglycylation) affects the ruggedness of the energy landscape for diffusion.^11^

Linear diffusion by proteins along double-stranded DNA is an important case of diffusion in a lower dimensional space and one that is crucial for proper cellular DNA processing. DNA-binding proteins (DBPs) perform various biological tasks, such as controlling transcription and repairing damaged DNA, all of which involve them scanning the DNA by linear diffusion prior to specific recognition at the functional site. Theoretical and experimental perspectives have attributed the remarkable efficiency and specificity of protein–DNA recognition to the 1D diffusion of proteins on DNA.^12−14^ Furthermore, diffusion along DNA has been observed experimentally for various DBPs, such as RNA polymerase,^15^ the *lac* repressor,^16−18^ the p53,^19−21^ restriction endonucleases,^22,23^ and Egr-1^24,25^ transcription factors, and for mismatch repair complexes, and its mechanisms have been further quantified by theoretical and computational studies.^17,26−47^

The proteins involved in DNA processing reactions have diverse structures. They may comprise different numbers of domains,^25,26^ have different oligomeric states,^48−50^ and exhibit intrinsically disordered regions to different extents.^51−53^ Furthermore, toroidal DBPs, being ring-shaped, encircle the DNA.^54^ It is assumed that many of these proteins, regardless of the differences in their structures, linearly diffuse along DNA. Nonetheless, the time spent engaged in linear diffusion along DNA may differ between proteins, as may the exact diffusion mechanism. These aspects may depend on their molecular characteristics and may be related to their function.

**Figure 1:**
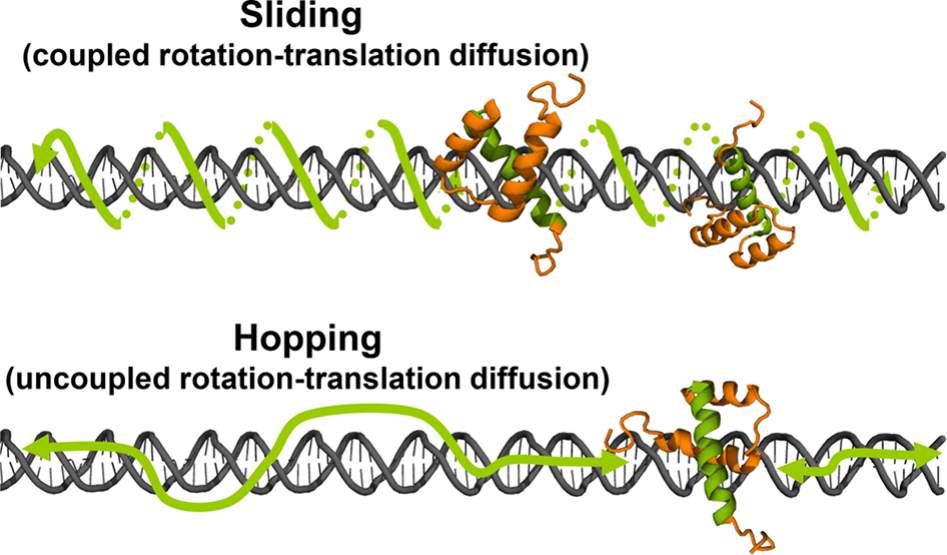
Schematic illustration of 1D diffusion of a DNA-binding protein along double-stranded DNA via the sliding and hopping mechanisms.

Linear diffusion may involve the stochastic translocation of the DBP predominantly along the longitudinal dimension of the DNA cylinder while its distance from the DNA axis varies depending on various factors, such as the salt concentration. This diffusion mechanism is often termed hopping dynamics (Figure 1). Alternatively, the higher nonspecific affinity of the DBP for the major DNA groove compared with other DNA sites (because of their electrostatic and shape complementarity) may allow the DBP to diffuse linearly and stochastically along DNA while its position is restricted by the DNA major groove. In this scenario, linear diffusion is characterized by coupling between translation and rotation. Such rotational–translational coupling is one of the main features of the 1D diffusion of proteins along DNA and is often termed sliding dynamics (Figure 1). As the electrostatic complementarity between DBPs and DNA is greater in the sliding mode than in the hopping mode, the usage of 1D diffusion via hopping is expected to increase as the salt concentration increases.^34^ Similarly, changing the DNA parameters by distortion or bending, for example, is expected to enhance hopping rather than sliding.^55,56^

The diffusion constant can be obtained from Einstein’s relation, *D = k*_B_*T*/ξ, where *k*_B_*T* is the Boltzmann constant multiplied by the absolute temperature and ξ is the friction for diffusion. For spherical particles ξ = 6Πη*R*, where η is the viscosity of the medium. This produces a Stokes–Einstein relation for translational diffusion of globular proteins of radius *R*, *D* = *k*_B_*T*/6Πη*R.* When the diffusion includes rotation, the friction follows ξ = 8Πη*R*^3^. The change in the dependence of *D* on the protein radius is often used to discriminate between hopping and sliding because the latter implies the existence of coupling between rotation and translational diffusion.^57,58^ The diffusion coefficients of various DBPs, which span 4 orders of magnitude (0.001–1 μm^2^/s), correlate better with 1/*R*^3^ than 1/*R*^1^, supporting the sliding mechanism. For some other proteins, the diffusion coefficients better correlate with 1/*R*.^59^ The implication of diffusion coefficient dependence on 1/*R*^3^ is that sliding is a slower diffusion mode compared with hopping for that protein. Additionally, a weaker dependence of *D* on salt concentration is often interpreted as an indication for use of the sliding rather than hopping mechanism.^22,34^

In this study, we focus on linear diffusion along double-stranded DNA. Using coarse-grained and atomistic simulations, we investigate the molecular driving forces of diffusion along DNA, focusing on how the structural and chemical features of DBPs and their environment affect the diffusion mechanisms the DBPs adopt and the requirements for DBPs to adopt the sliding diffusion mechanism characterized by rotational–translational coupling. We focus here on DBPs that comprise small binding domains as well as on the processivity factor sliding clamps, which serve as a unique example because of their toroidal topology.

## Methods

### Coarse-Grained Molecular Dynamics Simulations

The dynamics of protein diffusion along DNA was studied by using coarse-grained molecular dynamics (CG-MD) simulations that enable the investigation of long time scale processes that are challenging for high-resolution models. Each residue was represented by a single bead at the position of its Cα atom. The DNA was modeled with three beads per nucleotide, representing phosphate, sugar, and base.^34^

The force field applied in our simulations used a native-topology based model that included a Lennard-Jones potential to reward native contacts and a repulsive potential to penalize non-native contacts.^60,61^ Electrostatic interactions between charged residues (the bead representing the DNA phosphate groups bore a negative charge in our model) were modeled by using the Debye–Hückel potential.^61^

The explicit form of the force field is
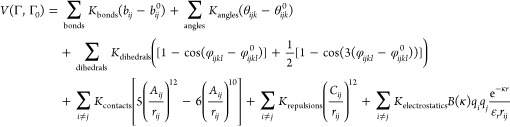
where *K*_bonds_ = 100 kcal mol^–1^ Å^–2^, *K*_angles_ = 20 kcal mol^–1^, and *K*_dihedrals_, *K*_contacts_, and *K*_repulsion_ are each valued at 1 kcal mol^–1^. The term *b*_*ij*_ is the distance (in Å) between bonded beads *i–j*, θ_*ijk*_ is the angle (in radians) between sequentially bonded beads *i–j–k*, φ_*ijkl*_ is the dihedral angle (in radians) between sequentially bonded backbone beads *i–j–k–l*, and *r*_*ij*_ is the distance (in Å) between beads *i–j* in a given conformation along the trajectory. *A*_*ij*_ is the distance (in Å) between beads *i–j* that are in contact with each other in the experimentally determined structure. The parameters denoted with the superscript 0 (*x*^0^) represent the minima of the various potential energy terms, which were assigned according to the atomic coordinates of the structures. *C*_*ij*_ is the sum of radii for any two beads not forming a native contact; the repulsion radius of the backbone bead is 2.0 Å. The last term in the force field is the Debye–Hückel potential, where *K*_electrostatics_ = 332 kcal Å mol^–1^ e^–2^, *q*_*i*/*j*_ is the sign of the charged residue, ε_r_ is the dielectric constant, κ is the screening factor, *B*(κ) is the salt-dependent coefficient, and *r*_*ij*_ is the distance (in Å) between charged residues *i* and *j*. We note that because of the coarse-grained representation of the systems, the effective salt concentration may correspond to a value higher (by a factor of ∼3) than for an atomistic representation. More details regarding the Debye–Hückel potential can be found in ref (61).

The dynamics of protein diffusion along DNA was simulated by using the Langevin equation. The simulation temperature was set to 0.4 (reduced units), which is lower than the folding temperatures of most of the studied proteins, namely, EB1, PRC1, SAP1, HD, HMG, and Skn1. The Tau protein, which was also studied, is intrinsically disordered and was simulated at the same temperature for consistency. The dielectric constant was 70, and the salt concentration was 0.01–0.06 M.

The DNA and diffusing protein were confined in a box of dimensions 300 × 300 × 300 Å^3^, and the longitudinal direction of the DNA was aligned along the *Z*-axis. We performed 10 simulations consisting of 10^7^ MD steps each. The DNA was modeled as a linear double-stranded B-DNA molecule with length 100 base-pairs. The diffusing DBPs and their protein data bank (PDB) identifiers (ID) were as follows: HD (PDB ID 1hdd), SAP1 (PDB ID 1bc8), Skn1 (PDB ID 1skn), human PCNA (PDB ID 5l7c, denoted herein as PCNA), and the HMG box (PDB 1hry). The diffusing microtubule-binding proteins were domains of EB1 (PBD ID 1pa7), PRC1 (PBD ID 5kmg), and Tau (PDB ID 6cvj).

To quantify the effect of point mutations on sliding, we designed a series of variants of a homeodomain DBP in which the number of positively charged residues at the recognition helix was varied, and the rest of the residues of the HD were neutralized. Specifically, we studied one mutant in which all six charged residues of the recognition helix remained charged, six mutants in which five residues remained charged, and 15 mutants in which four residues remained charged. For each case, the slope of θ/*Z* was estimated from 10 simulations sampled at a salt concentration of 0.01 M.

Three variants of PCNA were designed in which only a subset of the charged residues was included. In the first of the PCNA variants, all charges were neutralized in two of the three monomers, such that only one monomer bore the 24 positively and 38 negatively charged residues found in the wild type. In the second PCNA variant, all positive charges were neutralized in two of the three monomers such that only one monomer bore any positive charges, with the charged monomer having 24 positively charged residues. Finally, the charges on the third PCNA variant were neutralized to leave only six positive charges (residues K20, K77, K80, R149, H153, and K217) on one monomer. We refer to these mutants as the charged monomer variant, the positive monomer variant, and the six positive residues variant, respectively.

### Atomistic Simulations of PCNA

The trimeric PCNA protein ring used for the simulations was taken from the crystal structure available at the time (PDB ID 5L7C). All missing side chains in the three subunits were completed by using COOT (Crystallographic Object-Oriented Toolkit).^62^ The missing loop in each subunit in the 186–190 residue region was modeled by using Swiss-Model (ProMod3, v. 1.1.0,^63^). Protein Chain B of the trimer was used as the template. The resultant model was then used to complete the missing residues on the other two chains. A DNA dimer was built based on the sequence used by De March et al.^64^ in their simulations, with five base-pairs (GCGCG) added to each end, giving a 40-base-pair stretch of ideal B-DNA. The DNA was centered in the middle of the gap in the PCNA ring, and the ring was placed at the midpoint of the DNA chain, with the plane of the PCNA ring nearly perpendicular to the DNA axis.

The PCNA–B-DNA complex was placed in a dodecahedron box and solvated (tip3p water model). Sodium and chloride ions were added to a concentration of 0.125 M, adjusted to neutralize the overall charge of the system. The system was minimized and then equilibrated with the NVT and NPT protocols. Production runs of duration 2 μs were used for the analysis. Ten trajectories were sampled, with translation of PCNA along DNA observed in only six of them. We focus on these six trajectories. All simulations were performed with GROMACS package v. 2020^65^ and the AMBER99bsc1 force field.^66^

### Calculation of Diffusion Coefficients

The trajectories from the CG-MD simulations were analyzed by using in-house scripts. The mean-square displacements (MSD) of the proteins’ centers of mass (COM) were calculated via the equation
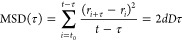
where *r* is the position of the protein COM, *t* is the number of time steps measured, and τ is the measurement window ranging from *t*_0_ to *t*. The slope of the MSD is 2*dD*, where *d* is the dimensionality of diffusion and *D* is the diffusion coefficient, which was calculated between time frames 1 and 200.

### Calculation of Rotation–Translation Coupling

The angle of rotation between the diffusing protein and the DNA was calculated in radians by , where *y* and *x* are the corresponding coordinates of the protein COM around the DNA, which was aligned along the *Z*-axis. For PCNA, θ was calculated based on the COM of one monomer. In the atomistic simulations, rotation of PCNA around DNA was measured as a dihedral angle based on three phosphate atoms on the DNA (G37 P, G33 P, and G23 P) and one α carbon on the protein (Asn200, chain A).

## Results and Discussion

The ability of proteins to diffuse along DNA via the sliding mechanism was studied here for a series of DBPs and non-DBPs by using CG-MD and atomistic MD simulations.

### Sliding of DNA-Binding Proteins along DNA

During sliding and while located at the major groove, a DBP retains continuous contact with the phosphates of the DNA backbone. In this case, a DBP will rotate 360° about the DNA approximately every 34 Å (10 base-pairs), which corresponds to the helical pitch of a canonical B-DNA molecule. Rotating along the helical path of the DNA enables the protein to continuously probe the base-pair content in the major groove of the DNA. To detect coupling between rotation and translation, we plotted the angle of the protein relative to the main DNA axis versus its translation along the DNA as measured in the coarse-grained simulations. A slope of 2π/34 = 0.18 rad/Å is indicative of diffusion involving rotation-coupled translation.

**Figure 2:**
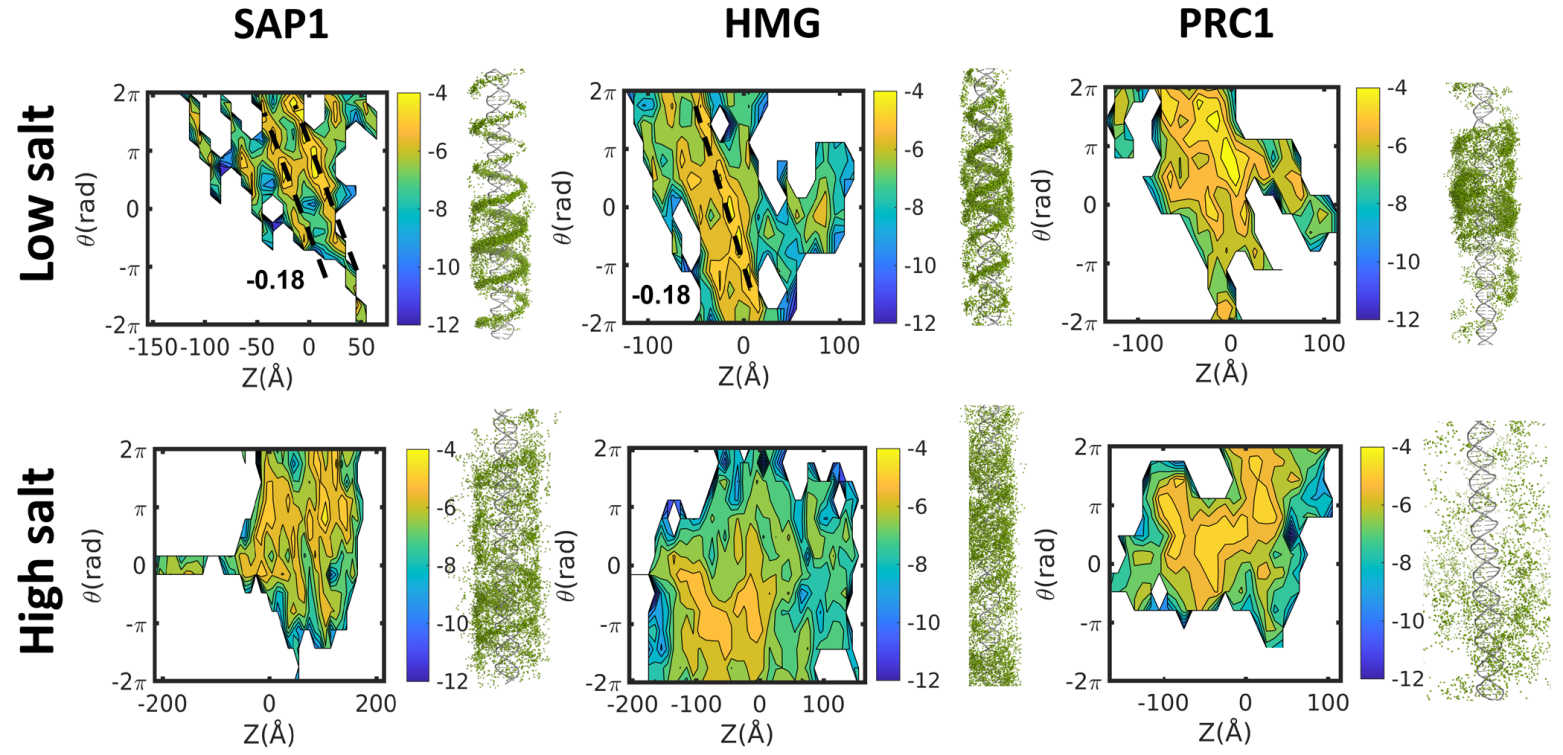
Coupling between rotation and translation as DNA-binding proteins diffuse linearly along DNA. The sliding mechanism is probed by projecting five sampled trajectories of three selected proteins in the [*Z*,θ] space, where *Z* indicates the location of the center of mass of the diffusing protein along the DNA axis and θ is the angle of the rotation performed by the protein. Projections are shown for the globular SAP1 DNA-binding protein, a minor groove HMG DNA-binding protein, and the PRC1 microtubule-binding protein at both low and high salt concentrations. Coupling between rotation and translation is revealed by a linear relationship between θ and *Z* with slope approximately −0.18 rad/Å. To the right, a cartoon representation of the projection of the binding-protein’s trajectory along the DNA is shown for each salt concentration condition.

We analyzed the mechanism of linear diffusion on DNA for several small-domain DBPs at a range of salt concentrations. Figure 2 (the projected trajectory on the right of each panel) pictorially illustrates the difference between the sliding and hopping mechanisms, sampled at low and high salt concentrations, respectively. We first examined the simulated linear diffusion along DNA of the SAP1 DBP, which binds the major groove of the DNA. At a low salt concentration (upper panel), tight coupling is observed between translation and rotation, and indeed the slope of θ/*Z* is −0.18 rad/Å for SAP1. This linear dependence between θ and *Z* is lost when SAP1 diffuses at higher salt concentrations (Figure 2, lower panel).

**Figure 3:**
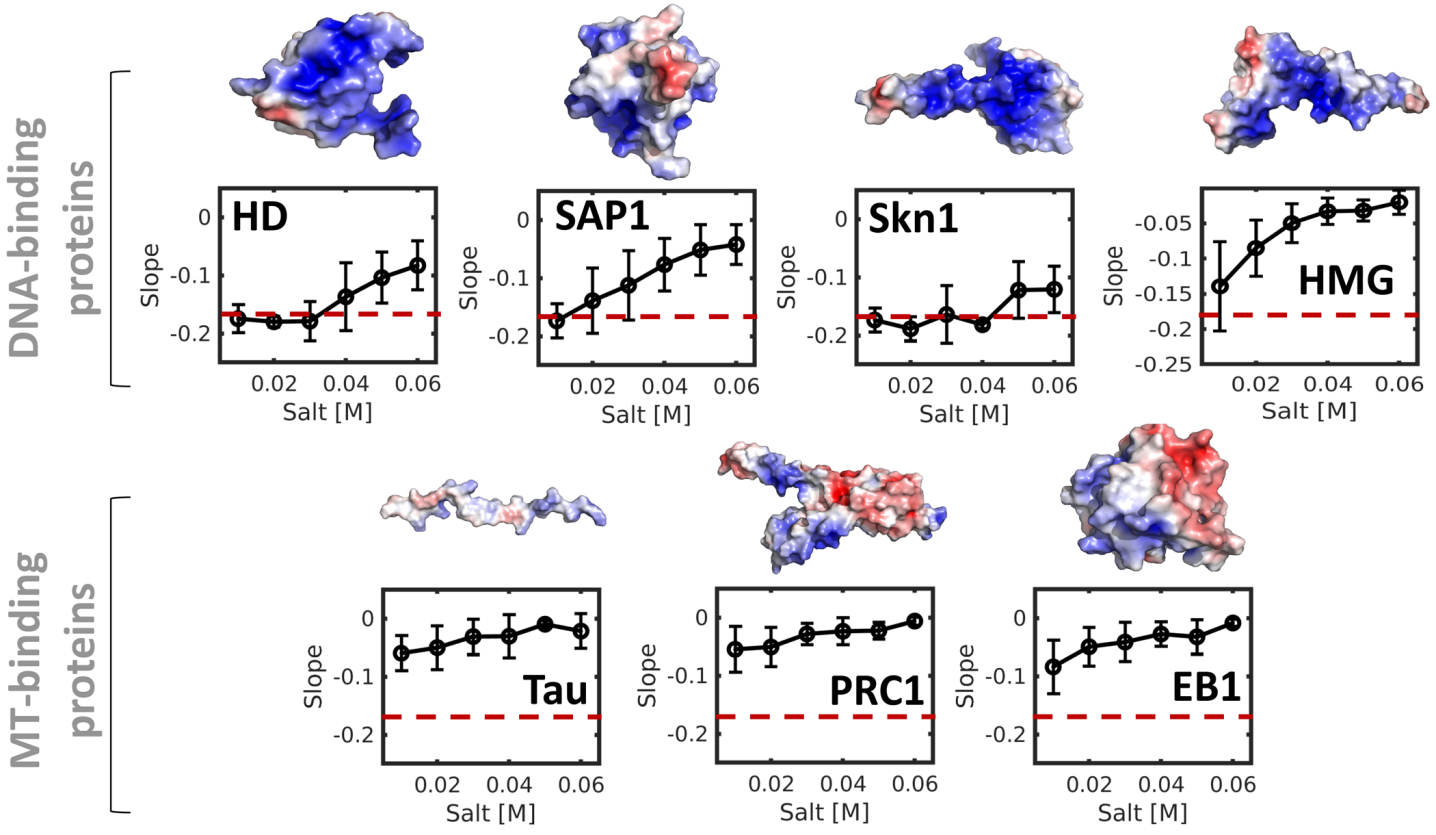
Coupling between rotation and translation during linear diffusion along DNA for various positively charged proteins: four DNA-binding proteins (HD, SAP1, Skn1, and HMG) and three microtubule-binding proteins (Tau, PRC1, and EB1). The electrostatic potential of each protein is shown on their corresponding structure with blue and red indicating positive and negative potential, respectively. Coupling between rotation and translation, which is indicated by slopes for the θ/*Z* plots of about −0.18 rad/Å, was measured at a range of salt concentrations and for both DNA- and microtubule-binding proteins. The error bars on the slope at each salt concentration were estimated from 10 independent simulations using the coarse-grained model. The value of the slope that corresponds to sliding is indicated by the red dashed line.

Figure 3 shows the dependence of the slope of a θ/*Z* curve on salt concentration for SAP1 as well as two other DBPs (the HD and Skn1 proteins). The mean slopes for these three DBPs illustrate that all of them slide on DNA at low salt concentrations. However, upon increasing the salt concentration, the slope increases from −0.18 rad/Å to values closer to 0 rad/Å, indicating a gradual transition from the sliding to hopping mechanism. The sensitivity of the diffusion mechanism to salt concentration varies between these three DBPs depending on their electrostatic potential.

Next, we explored how a DBP whose function involves binding to the minor groove diffuses along DNA. Figure 2 suggests that the HMG box can slide along DNA because the slope of its θ/*Z* plot is −0.18 rad/Å at low salt concentrations. However, Figure 3 shows that the sensitivity of the diffusion of the HMG box to salt concentration is larger than for the other DBPs, such that sliding constitutes a major diffusion mode for this protein solely at low salt concentrations.

**Figure 4:**
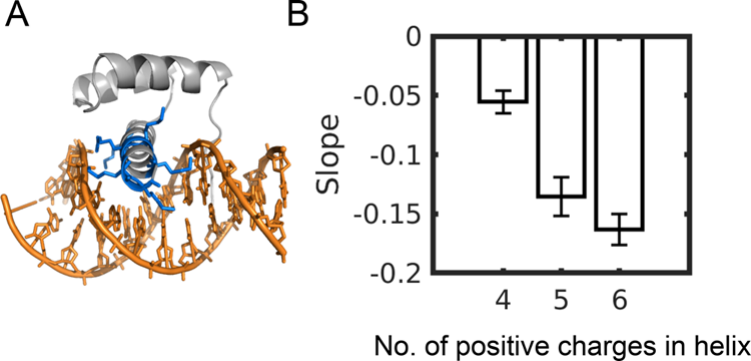
Effect of mutations on sliding along DNA. (A). Cartoon representation of the homeodomain (HD) protein bound to DNA, with its six positive residues in the DNA recognition helix colored in blue. (B). Value of the slope of a plot of θ/*Ζ* for the linear diffusion of HD mutants in which 0, 1, or 2 of the six positive residues in the recognition helix was neutralized to produce HD helices bearing 6, 5, or 4 positive charges, respectively. The error bars were estimated from the average of 10 trajectories for each variant. In addition, for HD variants that had only 4 or 5 charged residues, the averaging was also performed over 6 and 15 mutants, respectively.

The ability of three differently folded DBPs to slide along DNA via rotation-coupled translation suggests that this diffusion mechanism is common to many DBPs. This raises the question of what the minimal requirements are for a protein to slide along DNA. To address this question, we studied a series of variants of a homeodomain DBP in which the number of positively charged residues at the recognition helix was varied, and the rest of the residues of the HD were neutralized. Specifically, we studied one mutant in which all six charged residues of the recognition helix remained charged, six mutants in which five residues remained charged, and 15 mutants in which four residues remained charged. In each case, we considered all possible charge positions (Figure 4). We found that neutralizing two or more positive charges at any position on the recognition helix led to the loss of the characteristic rotation–translation coupled diffusion. Therefore, it appears that at least for the case of HD, a minimum of five positive charges at the recognition helix is necessary, albeit insufficient, for HD to slide along DNA via rotation-coupled translation.

### Sliding of Non-DNA-Binding Proteins along DNA

The finding that varied DBPs perform rotation-coupled translation along DNA suggests that electrostatics is fundamental to enabling such motion. The important role of electrostatics is revealed by the loss of coupling between rotation and translation upon mutating some charged residues. To further elucidate the role of electrostatics in linear diffusion and particularly its role at the onset of coupling between rotation and translation during diffusion along DNA, we studied the diffusion of proteins that normally interact electrostatically with microtubules (rather than DNA). Such an investigation can be valuable, given that microtubules are another negatively charged biopolymer^67^ and that the linear diffusion coefficients for microtubule-binding proteins sliding along microtubules are similar to the corresponding coefficients for DBPs sliding along DNA, that is, in the range 0.001–1 μm^2^/s depending on the protein’s dimensions.^67^

Figure 2 shows that the microtubule-binding protein PRC1 does not adopt rotation-coupled translation when it diffuses along DNA, even at a low salt concentration, as indicated by the lack of coupling between θ and *Z*. Simulating the diffusion of PRC1 and two additional microtubule-binding proteins, EB1 and Tau, along DNA at various slat concentrations highlights that their diffusion involves hopping at all the salt concentrations examined, as indicated by slopes of the θ/*Ζ* plots, which are close to zero (Figure 3). The origin of the inability of microtubule-binding proteins to slide along DNA is most likely related to their lower positive charge densities compared with DBPs.^67^ Their positively charged residues are sufficient for diffusion along the negatively charged microtubule, which is also complemented by the negatively charged C-terminal tails of the α- and β-tubulins. The nonspecific affinity of microtubule-binding proteins for DNA involves, therefore, diffusion that follows the hopping mechanism.

### Sliding of Toroidal Proteins along DNA

Toroidal proteins constitute an intriguing case study for diffusion mechanisms along DNA. Similarly to other DBPs, these ring-shaped proteins can linearly diffuse along DNA either by simple translation of the ring or by translation coupled with rotation. Nevertheless, their unique topology may impose some constraints. For example, ring-shaped proteins require disassembly of the ring to dissociate from DNA. Also, their internal symmetry may be compatible or incompatible with the DNA’s symmetry. Toroidal proteins that interact with DNA (e.g., helicases, topoisomerases, and some DNA repair proteins) are involved in various functions. A particularly pertinent case, when considering diffusion along DNA, is the toroidal sliding clamps that serve as processivity factors^68^ and assist other proteins to stay bound to DNA through multiple catalytic turnovers. The sliding clamps bind their respective DNA polymerase partner to template DNA, allowing it to replicate several bases without dissociating.^68^ The dynamics of sliding clamp diffusion along DNA is expected to be essential to its function.

The sliding clamps of eukaryotes and archaea are homotrimers called PCNAs (proliferating cell nuclear antigens).^69^ The sliding clamps do not have specific DNA-binding sites, but their circular assembly creates a positively charged channel in which duplex DNA can bind and slide freely. The inner diameter of the clamp is about 30 Å, compared to the 20 Å diameter of duplex DNA. The microscopic details of the interactions between the inner ring of the clamp and the DNA may dictate the sliding mechanism. The combined consequences of the topological constraints imposed on the DNA by its localization within the inner ring together with the extensive and symmetric electrostatically attractive forces are not trivial.

PCNA is the most-studied sliding clamp, and several studies have been conducted to assess the diffusion mode it adopts while diffusing along DNA. These studies, which include simulations^70^ and single molecule imaging^71,72^ as well as X-ray crystallography,^64^ have yielded some ambiguous results. Single-molecule studies showed that increasing the size of PCNA by attaching a quantum dot resulted in a very mild decrease in *D*, which is suggestive of the hopping mechanism.^73^ However, the same study reported that changing the solution viscosity had little effect on *D*, which was argued to support sliding.^73^ Furthermore, the linear diffusion coefficient of PCNA showed, both experimentally^73^ and computationally,^74^ a weaker dependence on salt concentration than is often found for globular DBPs. While this observation can be regarded as implying use of the sliding mechanism, such an inference may not apply to a toroidal protein that encircles the DNA and thus cannot dissociate from the DNA under conditions of increased salt concentration unless it first disassembles. Similarly, mutating the charged residues had much smaller effect on diffusion speed along DNA for PCNA compared with transcription factors. This suggests that electrostatics play a smaller role in the linear diffusion of PCNA, consistent with the hopping mechanism.^25,34,49^

A recent study of PCNA sliding that used X-ray crystallography, NMR spectroscopy, and molecular dynamics simulations supported the existence of rotation-coupled translation, which occurs when the ring-shaped PCNA protein is tilted relative to the DNA axis.^64,75^ The strength of the coupling between the rotation and translation of sliding clamps as they diffuse along DNA, and other molecular determinants that govern their diffusion, require further quantification, and we therefore examined them using both our CG-MD model and atomistic simulations.

### Coarse-Grained Molecular Dynamics Simulations of PCNA Diffusion along DNA

**Figure 5:**
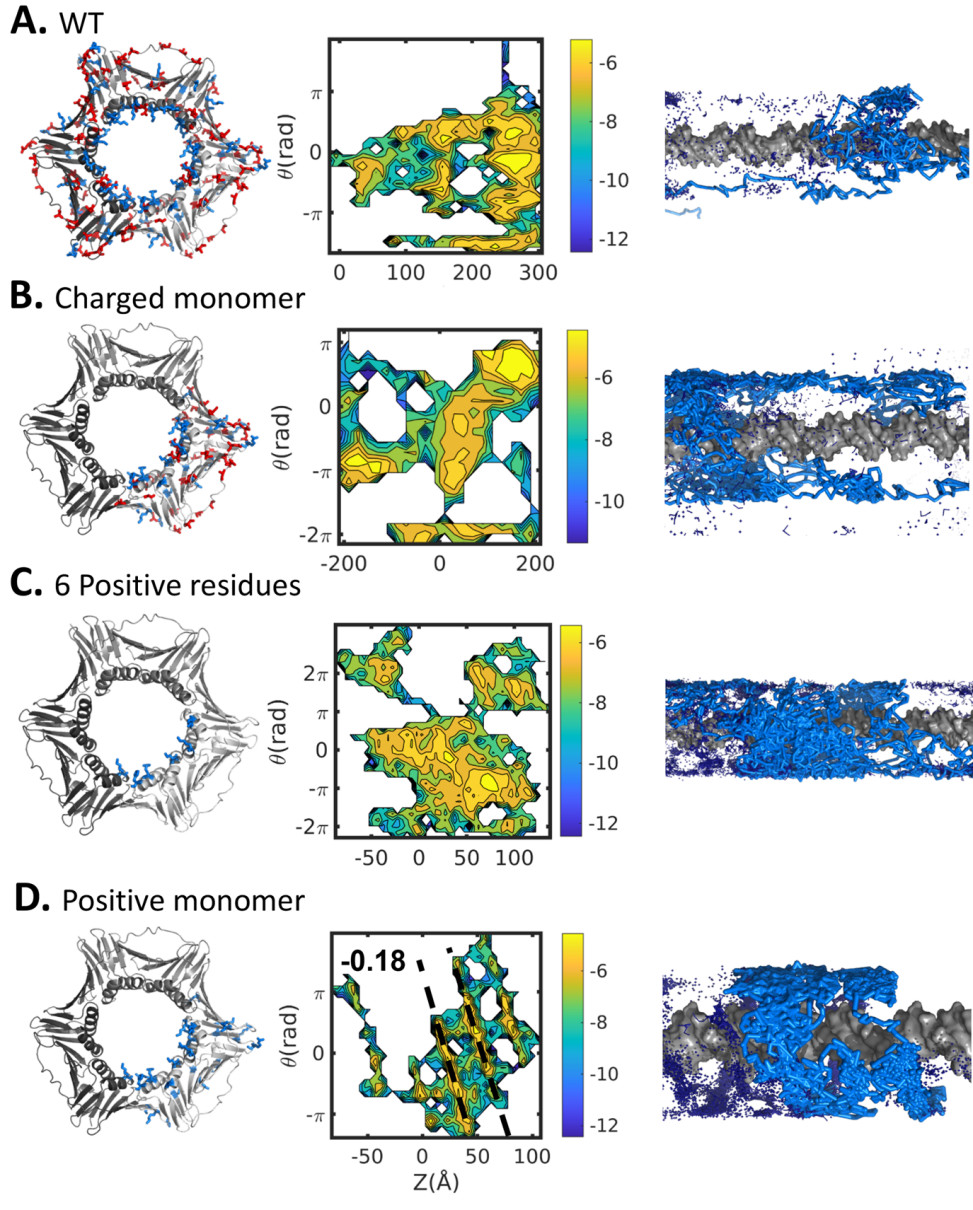
Coupling between rotation and translation as PCNA diffuses linearly along DNA. The diffusion mechanism is examined for (A) WT PCNA homotrimer and (B–D) three variants in which only a subset of the WT charges was included with all other charges neutralized. In the PCNA variants, (B) all charges were neutralized in two of the three monomers, leaving one charged monomer (the “charged monomer” variant); (C) all charges were neutralized on two of the monomers and all negative and most positive charges were neutralized on the remaining monomer, such that six positive charges remained on that monomer (the “6 positive residues” variant); and (D) all positive charges were neutralized from two of the three monomers such that only one monomer bore positive charges (the “positive monomer” variant). The leftmost panels show each toroidal PCNA protein variant, with blue and red indicating positive and negative potential, respectively. The middle panels show projections of the five sampled trajectories simulated by using the coarse-grained molecular dynamics model in the [*Z*,θ] space, where *Z* indicates the location of the center of mass of one monomer of the diffusing protein along the DNA axis and θ is the angle of the rotation performed by the protein. The rightmost panels present cartoon representations of the diffusion mechanisms. Sliding is evident only for the positive monomer variant (D).

Molecular dynamics simulations using coarse-grained models showed that PCNA does not follow the DNA major groove when it diffuses along DNA. Although PCNA can rotate around the DNA, the speed of its translocation along the DNA, however, is much greater than that obtainable by rotation. Accordingly, the diffusion of PCNA along DNA is not necessarily rotation-coupled translation. To examine PCNA’s ability to utilize the rotation-coupled translation mechanism to diffuse linearly along DNA at a low salt concentration, we employed the CG-MD model and plotted rotation angle versus translocation for the wild type and three mutants. The plot shown in Figure 5A indicates no coupling between rotation angle and translation for wild type (WT) PCNA and that its linear diffusion on DNA is consistent with the hopping mechanism.

The adoption of hopping dynamics is consistent with the weaker electrostatic interface that PCNA forms with nonspecific DNA in comparison with the interfaces formed by other DBPs. The reported *K*_D_ for PCNA–DNA is 0.7 mM,^64^ whereas the corresponding values for other proteins are a few micromolar.^76^ The dynamic nature of the PCNA–DNA interface is also supported by the high *B*-factors of the DNA in its crystallized complex with PCNA.^64^ Indeed, our previous coarse-grained study of PCNA showed that during linear diffusion the DNA tends to remain close to the central axis of the inner PCNA cavity.^74^ Accordingly, the PCNA–DNA interface is frustrated by the electrostatic forces between the ring and the cylindrical DNA. Given the impossibility of satisfying all the potential electrostatic interactions simultaneously, the lowest energy is achieved when the DNA is located at the center of the ring. It is possible that this electrostatic frustration and the imperfect geometrical fit of the DNA within the inner ring of the PCNA result in fast linear diffusion that shows only weak coupling between rotation and translocation.

Coarse-grained simulations are very powerful tools for the study of variants that are difficult to study experimentally. Accordingly, in addition to WT PCNA, we studied variants of PCNA that were designed to examine the minimal requirement for PCNA sliding along DNA. Three additional variants of PCNA were designed in which only a subset of the charged residues was included and shown schematically in Figure 5 (see also the Methods section). Neutralization of two of the three PCNA subunits to produce a mutant with only one charged monomer still did not produce coupling between rotation and translation, as indicated by a lack of linear correlation in plots of θ vs *Z* in the projected simulations (Figure 5B).

**Figure 6:**
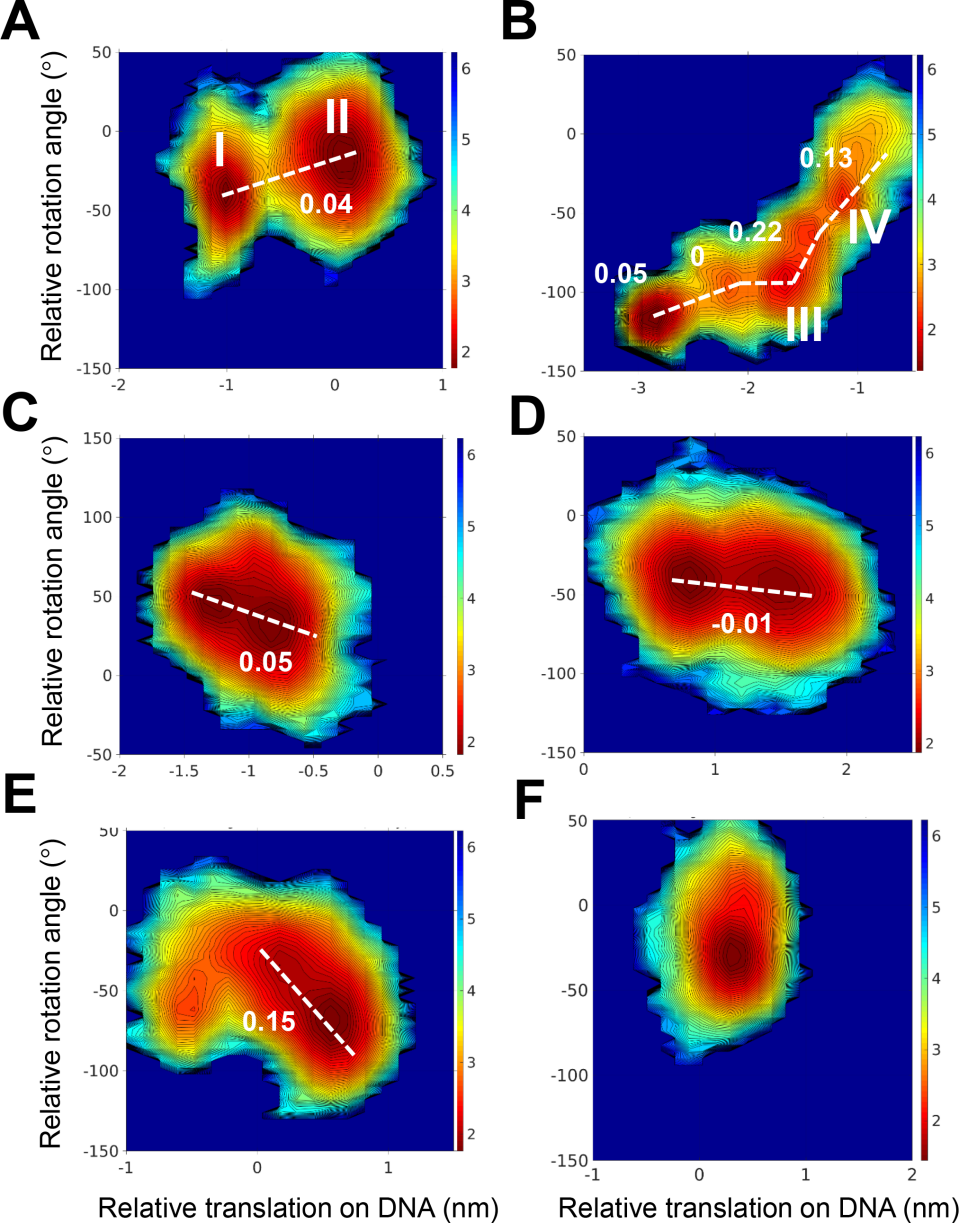
Coupling between rotation and translation of PCNA during linear diffusion along DNA.( A–F). Six 2 μs atomistic trajectories of PCNA diffusing along DNA projected in the [*Z*,θ] space. The slopes observed in each trajectory are indicated by a white dashed line together with their corresponding values (in rad/Å). The trajectory represented in panel F does not show significant translation, and so no slope was measured. In each of the trajectories projected in panels A and B, two states are highlighted (I and II in panel A; III and IV in panel B) which are further analyzed in Figure 7.

Although the coarse-grained simulations show no coupling between rotation and translation for the linear diffusion of WT PCNA along DNA (Figure 5A), which is supported by various experimental findings, the X-ray structure of the PCNA–DNA complex (PDB 6GIS or its originally deposited ID 5L7C) suggested the presence of an interface defined by six hydrogen bonds between PCNA residues K20, K77, K80, R149, H153, and K217 and the DNA. It was suggested that these hydrogen bonds involve five consecutive phosphates of a single DNA strand. Although the quality of the electron density in this crystal structure was questioned recently,^77^ we examined whether the reported pattern of hydrogen bonding may support rotation-coupled translation, as was conjectured on the basis of this structure. We found that the PCNA variant that includes only these six positive residues on a single subunit (while all the other charges in PCNA are neutralized) does not slide along the DNA (Figure 5C). However, when all the positive charges of a single subunit are included in the coarse-grained simulations, the diffusion follows a sliding mechanism (Figure 5D).

### Atomistic Simulations of PCNA Diffusion along DNA

To undertake a high-resolution assessment of whether PCNA sliding can also occur for WT PCNA and not only for the variant with a positively charged subunit, we simulated WT PCNA using an atomistic MD model. We performed six simulations, each of duration 2 μs. Figure 6 shows projections of each of the atomistic simulations in the [*Z*,θ] space. As expected, these simulations show much more limited diffusion compared with that observed in the coarse-grained simulations. The translation identified during the 2 μs time scale is between 5 and 25 Å. In most simulations (Figure 6A–E), at least two major states are populated during the diffusion performed by the PCNA. These two states were used to measure the slope of the θ/*Ζ* plot. One simulation produced limited translation with only a single populated state; therefore, a slope was not estimated in this case (Figure 6F). The slopes have different values that indicate diverse diffusion mechanisms. In three simulations (Figures 6A, 6C, and 6D), the diffusion of PCNA is defined by a slope of −0.05–0 rad/Å and thus corresponds to a hopping mechanism. In two other trajectories (Figures 6B and 6E), the slope, at least in part of the simulations, is 0.15–0.2 rad/Å and may support rotation-coupled translation diffusion.

**Figure 7:**
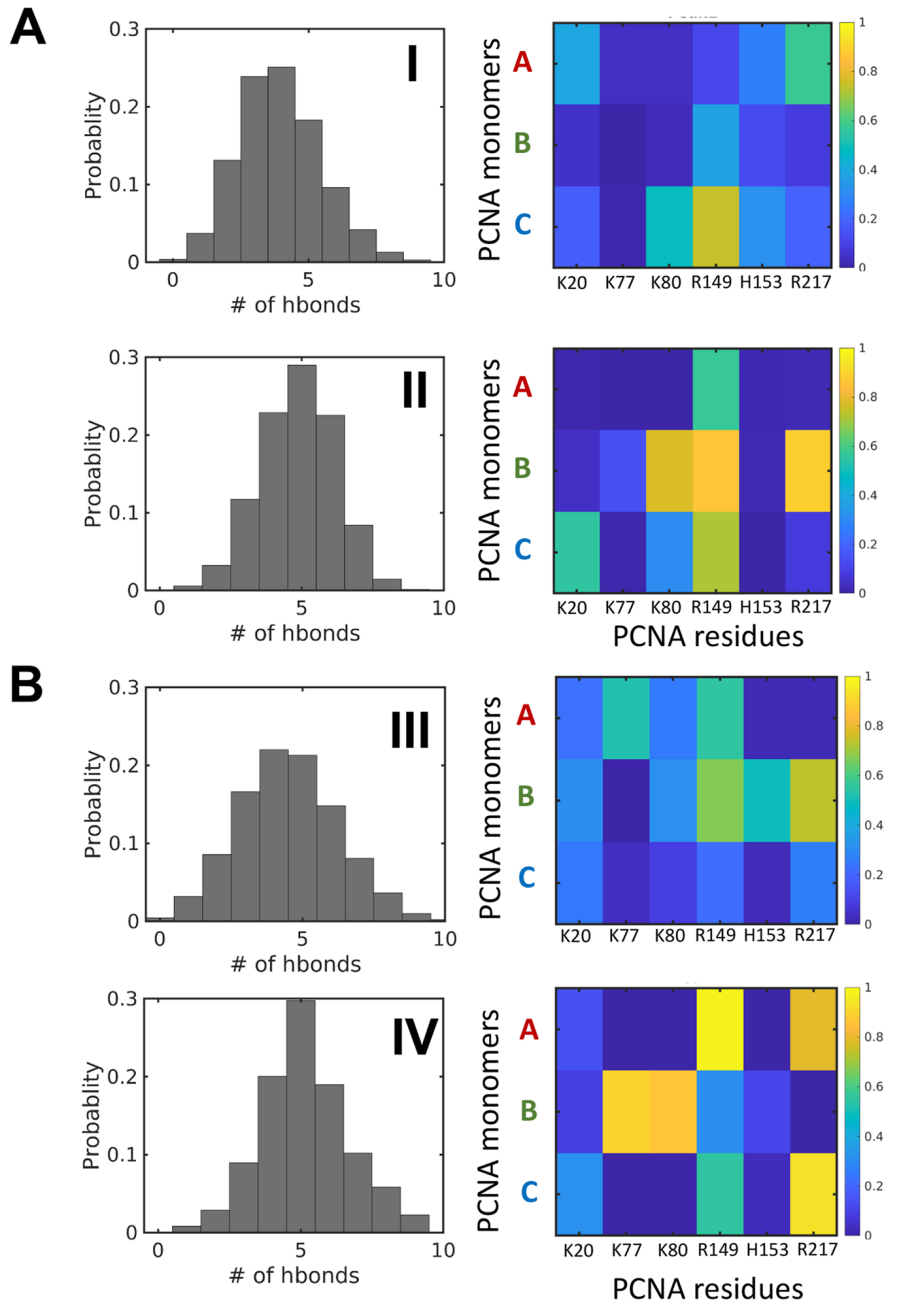
Hydrogen bonds formed between PCNA and DNA. (A and B) Hydrogen bond analysis for the trajectories shown in Figures 6A and 6B, respectively. The number of hydrogen bonds formed between PCNA and DNA in these trajectories is depicted by histograms. Two states in each trajectory are analyzed: in trajectory A, states I and II, and in trajectory B, states III and IV (see Figure 6). The PCNA residues involved in these hydrogen bonds are analyzed in the matrices, which show the formation probability of each hydrogen bond and the location of the positive residues in monomers A, B, or C of the PCNA trimer.

To explore the mechanism of the linear diffusion of PCNA along DNA, we analyzed the hydrogen bonds formed between PCNA and DNA in the two sampled trajectories that exhibit the largest dynamics (those shown in Figures 6A and 6B). Figure 7 shows the distribution of the total number of hydrogen bonds in the major states of each trajectory (labeled in Roman numerals as per Figures 6A and 6B). In each state, the number of hydrogen bonds that define the interactions between PCNA and DNA is quite broad, indicating that the system is dynamic even when no major translocation is measured. For example, for trajectory A (Figure 7A, corresponding to the simulation projected in Figure 6A), the number of hydrogen bonds is 1–9 (mean = 6) throughout the simulations. This is consistent with the highly dynamic nature of the PCNA–DNA interface, as suggested by its high *B*-factor, and strengthens the suggestion that the PCNA–DNA interface is dynamic and that transient salt bridges can be formed simultaneously between the DNA cylinder and all subunits of the ring of PCNA.

When analyzing which residues participate in these hydrogen bonds, we noticed that some of the residues are involved in hydrogen bonds between PCNA and DNA in the two main states (i.e., I and II or in III and IV), yet at different probabilities. One of the main characteristics of the hydrogen-bonding pattern is that these bonds do not form between a single monomer of PCNA and DNA. In the two trajectories between the four identified states, all three PCNA monomers interact with DNA, which suggests that the DNA lies at the center of the inner ring of the PCNA. This pattern of hydrogen bonds, to which different PCNA subunits contribute, is observed regardless of the diffusion mechanism adopted, including when the θ/*Ζ* slopes are closer to the value of −0.18 rad/Å which corresponds to rotation-coupled translation diffusion.

**Figure 8:**
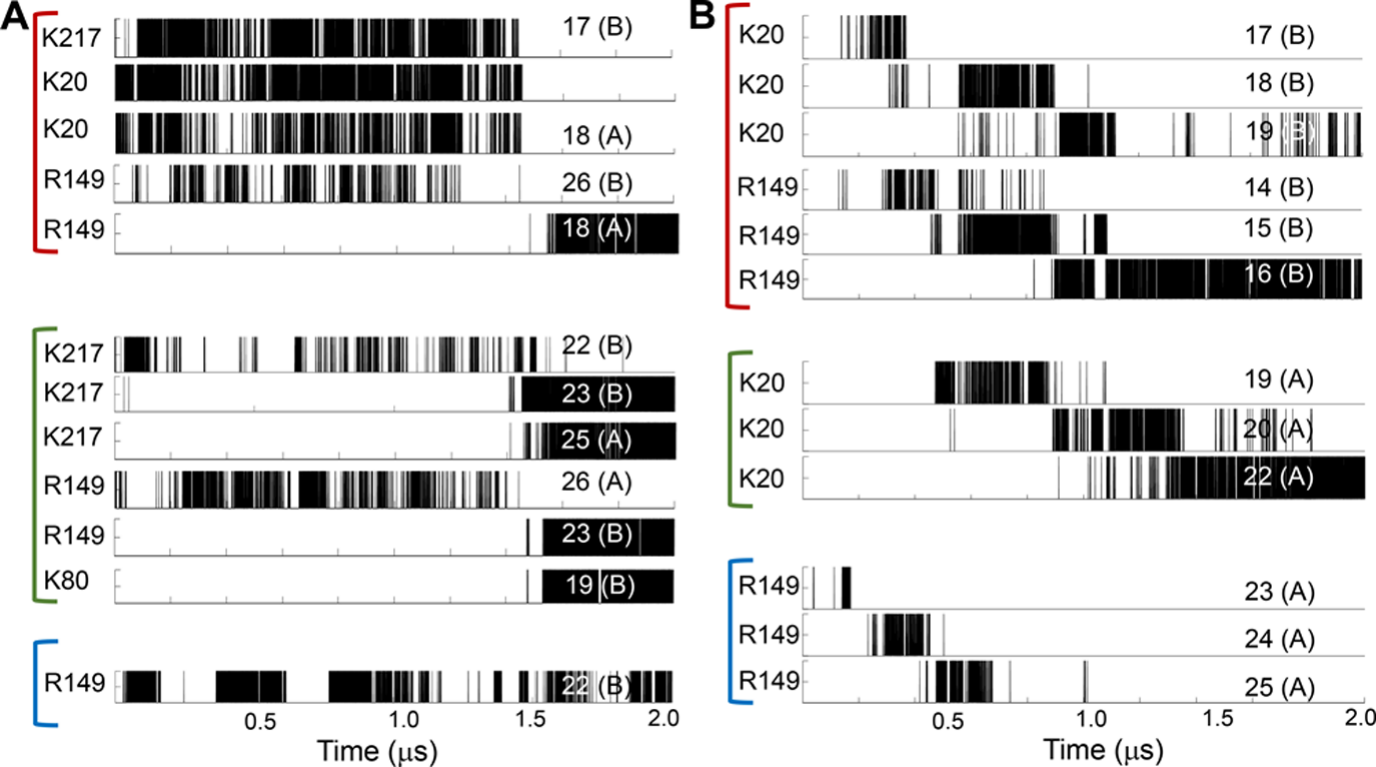
Time evolution of hydrogen bond formation during diffusion of PCNA along DNA. Hydrogen bonds formed between PCNA residues and DNA nucleotides during 2 μs trajectories of the diffusion of PCNA on DNA. Hydrogen bonds formed between some selected PCNA residues and DNA nucleotides were tracked for the trajectory (A) shown in Figure 6A and trajectory (B) shown in Figure 6B. Residues from PCNA monomers A, B, and C are grouped by red, green, and blue lines, respectively. The identities of the PCNA residues and the DNA nucleotides that participate in each of the probed hydrogen bonds are indicated at the left and right of each panel, respectively. The identity of the DNA strand participating in each hydrogen bond is indicated in parentheses by either A or B.

To understand the diffusion mechanism better, we followed the time evolution of selected hydrogen bonds formed in trajectories A and B (Figure 8). Figure 8A illustrates that hydrogen bonds involving residues from all three PCNA monomers and the DNA are involved in diffusion. These hydrogen bonds do not follow a particular pattern of breakage and formation, which supports adoption of the hopping mechanism, as is also suggested by the θ/*Z* slope of −0.04 rad/Å (Figure 6A). Similarly, Figure 8B also shows that residues from all three monomers are involved in hydrogen bonds with nucleotides from both DNA strands. However, in this trajectory the hydrogen bonds show a gradual shift to subsequent nucleotides, so supporting sliding dynamics, consistent with the greater slope (Figure 6B).

## Conclusions

Diffusion is a common transportation mechanism in the cell, particularly when it takes place in lower dimensionality spaces, such as proteins diffusing along 1D biological polymers. Examples of 1D diffusion include the dynamics of proteins along DNA or along microtubules. These diffusion processes are essential for proper cellular function. Although each diffusion mechanism potentially has unique characteristics, they share some common features, such as the role of long-range electrostatic forces in mediating diffusion.^5,10,34,78^

In this study, we examined protein diffusion along DNA while the proteins search for their cognate site. Many proteins are known to be able to diffuse helically along the major groove, which may enable them to probe the DNA sequence and subsequently to bind specifically to their target sites. Here, we asked what the requirements are for helical diffusion along DNA and under what conditions the 1D diffusion will follow simple linear translation (i.e., hopping) instead of rotation-coupled translation (i.e., sliding).

The ability of globular DBPs to slide along DNA while situated at the major groove is related to both electrostatic and structural complementarities. Reducing the electrostatic strength by increasing the salt concentration may shift diffusion on DNA from sliding to hopping. Similarly, DBP mutations that reduce their charge density may result in diffusion via hopping, but this strongly depends on the location of the mutations. For the homeodomain, we found that the presence of five positively charged residues in the recognition helix is essential for sliding dynamics whereas the presence of a smaller number of charged residues may result in hopping. We found that a DBP that interacts at the DNA minor groove can also slide when it is placed at the major groove. Sliding appears to be a mechanism common to diverse DBPs that possess different structural features and perform various functions. However, the detailed biophysical features of sliding dynamics can be different for different globular DBPs. The diffusion of DBPs along DNA may vary, for example, with respect to the durations of uninterrupted sliding events (before they are interrupted by hopping or dissociation events), by the lengths of DNA that are scanned in each sliding event, and by the 1D diffusion coefficients.^79^

Several DBPs have been characterized experimentally to diffuse on DNA in a nonhelical fashion (i.e., by hopping). These proteins include the TALE^59^ and the processivity factor UL42^80^ proteins. Possible reasons for the lack of sliding may be their weak electrostatic affinity as well as structural and topological features that conflict with the rotation-coupled translation mechanism. Toroidal DBPs serve as interesting cases because, although their symmetric ring shape enables them to rotate around the DNA, it is unclear whether this rotation can couple with translation. A particularly interesting toroidal protein is the PCNA, regarding which there is controversy as to whether its diffusion is via sliding or hopping. Some earlier efforts endeavored to quantitatively characterize the diffusion of PCNA along DNA from the experimental and computational^70^ perspectives. Our coarse-grained simulations show that WT PCNA diffuses by means of decoupled translation and rotation. Only a variant that includes the positively charged residues in a single subunit exhibits sliding dynamics. The atomistic simulations, while capturing limited diffusion, are insightful as they show that the diffusion of WT PCNA often follows hopping dynamics; however, one trajectory among the six sampled trajectories supports sliding dynamics. Another recent atomistic simulation using a different force field also reports that the diffusion of PCNA does not involve coupling between rotation and translation.^81^

The interaction between PCNA and DNA is stabilized by 1–9 hydrogen bonds. In all the sampled events, these hydrogen bonds involve residues from all three PCNA subunits interacting with DNA, which can be caused by localization of the DNA in the center of the PCNA ring or by tilting of the ring with respect to the DNA axis. In either case the DNA does not interact with a particular PCNA subunit for any extended period. This result opposes the interpretation of a recent structural study that the five hydrogen bonds between residues are localized in a single subunit of PCNA interacting with DNA,^64^ resulting in significant coupled rotation/translation events. This X-ray structure was criticized for its lack of electron density for DNA.^77^ We note that a recent cryo-EM structure of PCNA with DNA in the presence of DNA polymerase δ supports our finding that the DNA is located in the center of the PCNA inner ring.^82^

In summary, our study complements previous studies highlighting the widespread nature of rotation-coupled diffusion of proteins along DNA. Although rotation-coupled translation was reported for many proteins, for some others it was excluded.^59^ Recently, it was shown that two DNA-repair proteins with similar structures both slide at low salt concentrations, but one of them follows mostly the hopping mechanism at higher salt concentrations,^83^ which might be linked to its function. The accumulated results so far thus suggest that protein structure, topology, and electrostatic potential together with the electrostatic potential of the DNA conformations^56^ may modulate the balance between the usage of sliding and hopping mechanisms for the linear diffusion of proteins along DNA.
